# Ultrasmall Manganese Nanospinels Produced via an Alcohol Reduction Method and Their Electrocatalytic Oxygen Evolution Reactivity

**DOI:** 10.1021/acsami.4c18777

**Published:** 2025-04-07

**Authors:** Yuuki Sugawara, Kazuyuki Iwase, Reona Iimura, Takashi Yabu, Akira Nasu, Masaki Matsui, Itaru Honma, Takeo Yamaguchi, Hiroaki Kobayashi

**Affiliations:** † Laboratory for Chemistry and Life Science, Institute of Integrated Research, Institute of Science Tokyo, R1-17, 4259 Nagatsuta-cho, Midori-ku, Yokohama, Kanagawa 226-8501, Japan; ‡ Institute of Multidisciplinary Research for Advanced Materials, Tohoku University, 2-1-1 Katahira, Aoba-ku, Sendai, Miyagi 980-8577, Japan; § Department of Chemistry, Faculty of Science, Hokkaido University, Kita 10, Nishi 8, Kita-ku, Sapporo, Hokkaido 060-0810, Japan

**Keywords:** water electrolysis, electrocatalyst, oxygen evolution reaction, metal oxide, nanospinel, elemental effect

## Abstract

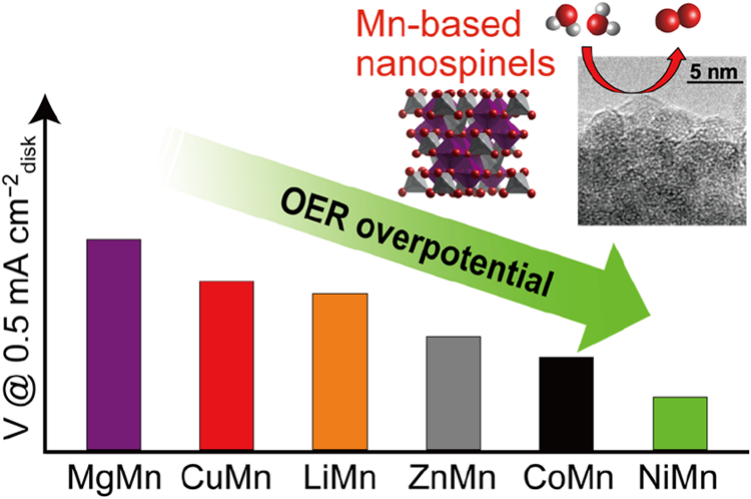

Green hydrogen production via electrochemical water splitting extensively demands the development of cost-effective and highly efficient electrocatalysts for the anodic oxygen evolution reaction (OER). Nanosized spinel nanoparticles (nanospinels) are potential candidates as electrocatalysts for the OER because of their very high specific surface areas. This work systematically investigated the influence of the A-site metals in the Mn-based nanospinels, i.e., LiMn_2_O_4_, MgMn_2_O_4_, ZnMn_2_O_4_, NiMn_2_O_4_, CuMn_2_O_4_, and CoMn_2_O_4_. Evaluation of their OER activities then indicated that higher OER activities for NiMn_2_O_4_ and CoMn_2_O_4_ than those of ZnMn_2_O_4_, LiMn_2_O_4_, CuMn_2_O_4_, MgMn_2_O_4_, and NiMn_2_O_4_ nanospinels possessed the best OER activity among the Mn-based nanospinels. Additionally, the NiMn_2_O_4_ nanospinel exhibited a dramatically improved OER performance compared with NiMn_2_O_4_ synthesized by the conventional sol–gel process with a much larger particle size, which indicated the advantage of employing nanospinels as an OER electrocatalyst. Also, the NiMn_2_O_4_ nanospinel was one of the best OER electrocatalysts among the previously reported bimetal spinel oxides. Finally, operando XAFS measurements using an in-house electrochemical cell unveiled that the surface of the NiMn_2_O_4_ nanospinel was electrochemically transformed to Mn–Ni hydroxide under an OER potential, and the generated compound was preferable for the OER process. This work uncovered that nanospinels are promising candidates as OER electrocatalysts and provided a guideline for the selection of metal components for nanospinel design.

## Introduction

Paving the way to a worldwide sustainable energy system for conserving the environment and preventing global warming is the most challenging task for the human society. Especially, with an explosion in population and the scarcity of conventional energy resources, such as fossil fuels, turning to alternative renewable energies, e.g., solar and wind power, is highly demanded. To efficiently use renewable energy-generated electricity, the future sustainable human society desires efficient electrochemical energy conversion and storage technologies, such as lithium^1−3^ and other ion batteries,^4,5^ fuel cells,^6,7^ electrochemical ammonia synthesis,^8^ electrochemical CO_2_ reduction, and hydrogen production via water splitting.^9,10^ To actualize this scenario, improvement of electrode materials with sufficient efficiency and selectivity is considerably crucial. Therefore, a large number of studies have demonstrated the development of electrode materials for different energy conversion/storage reactions, including experimental, theoretical, and data-driven approaches.^11^

Hydrogen production with high purity from abundant water using renewable energy-derived electricity is one of the most promising candidates for future energy systems due to the high energy density of hydrogen and its lack of emissions. Thus, water splitting technology has drawn considerable interest as a clean and cost-effective energy conversion platform for a future sustainable society. However, a major bottleneck in water splitting is the sluggish oxygen evolution reaction (OER) at the anode side, which prevents the application of this technology to the power-to-gas approach as an efficient energy conversion platform. Various types of electrocatalysts have been studied to promote the OER, but well-known active compounds are mostly composed of scarce precious metals, e.g., IrO_2_ and RuO_2_.^12−14^ Therefore, alternative OER catalysts bearing earth-abundant main-group and/or transition metals are highly desirable, and many efforts in the past decade have been intensely devoted to developing intrinsically active OER catalysts with earth-abundant metals for alkaline water splitting^15−24^ because such nonprecious metals are thermodynamically stable in alkaline conditions.

Among nonprecious metal-based OER catalysts, spinel oxides AB_2_O_4_, where tetrahedral A- and octahedral B-sites are occupied by main-group or transition metals and transition metals, respectively, have recently attracted much interest and exhibited excellent electrocatalytic activity for the OER in alkaline media.^25−27^ For example, several Mn-based spinels have previously been reported as promising OER electrocatalysts in alkaline media.^28−30^ Additionally, we recently reported the structure-dependent OER activities of CoMn_2_O_4_ analogues^31^ and successfully demonstrated a prominent OER performance by directly anchoring the Co–Mn catalysts on a graphene sheet.^32^ The enhanced activity by anchoring on the graphene sheet was attributed to its enlarged specific surface area due to the minimization of catalyst particles, i.e., the decrease in the particle size from 200–300 to 40–80 nm resulted in 66 times higher mass-normalized OER activity.

Recently, efficient synthetic routes for spinels with single nanometer-sized particle diameters (nanospinels) were developed based on an unprecedented synthetic route. The synthesized nanospinels exhibited a prominent electrochemical performance as electrodes for energy storage devices. For example, sub-5 nm MgCr_2_O_4_^33^ and MgMn_2_O_4_ with ≈10 nm of particle size^34^ exhibited excellent capacity as a cathode material in Mg ion batteries. Our group also reported ultrasmall and ultraporous Mn spinels (MgMn_2_O_4_,^35^ CuMn_2_O_4_,^36^ and ZnMn_2_O_4_^37^) synthesized by an in-house alcohol reduction method under room-temperature conditions. This MgMn_2_O_4_ nanospinel demonstrated excellent efficiency for cathode reactions in Mg ion batteries. In addition to the battery electrodes, nanospinels are also potential candidates as electrocatalysts for water splitting because their single nm-sized particles lead to very high specific surface areas (>100 m^2^ g^–1^). The high surface area results in abundant reaction sites and is expected to dramatically enhance the electrocatalytic mass activity, which is defined as a catalytic current normalized by the loading mass of the catalysts on the electrode and is crucial for practical implementation in terms of cost-effectiveness. For instance, OER electrocatalysis on nanospinels was investigated with its carbon nanotube (CNT) composite.^20^ It is well known that the OER activity of spinels can be significantly tuned by substituting and doping on the A- and B-sites.^38−40^ Therefore, systematic control of their particle size and elemental composition should be a reasonable approach to elucidating their OER electrocatalysis on nanospinels. The above-mentioned alcohol reduction method for nanospinel synthesis is unprecedented and can selectively fabricate a wide range of nanospinels with several metallic compositions; however, there exists no systematic study to clarify the OER electrocatalysis of the Mn-based nanospinels with several tetrahedral A-sites.

Herein, we aim to uncover the influence of the metallic elements in the tetrahedral A-sites in the Mn-based nanospinels on their OER electrocatalysis under alkaline conditions by experimental analyses. We examine three main-group metals, Li, Mg, and Zn, and three transition metals, Ni, Cu, and Co, which are our previously reported CoMn-based nanospinels^31^ as A-site metals. We synthesize the series of the above-mentioned Mn-based nanospinels, i.e., LiMn_2_O_4_, MgMn_2_O_4_, ZnMn_2_O_4_, NiMn_2_O_4_, CuMn_2_O_4_, and CoMn_2_O_4_ via similar processes and evaluate their OER activities under identical conditions. Finally, we perform operando X-ray absorption measurements to elucidate the structural and valence states of the nanospinel under the OER process to provide insights into its OER electrocatalysis. This work can give a systematic study of the influence of the A-site metals in Mn-based nanospinels on their OER electrocatalytic activity and aim at providing a guideline for the selection of metal components in OER electrocatalysts.

## Materials and Methods

### Chemicals

Ni(NO_3_)_2_·6H_2_O (99.9%), Mn(NO_3_)_2_·6H_2_O (99.9%), and potassium hydroxide (KOH, 85%) were purchased from Wako Pure Chemical Industries, Ltd. (Osaka, Japan). Citric acid (99.0%) was purchased from Kanto Chemical Co., Inc. (Tokyo, Japan). Five wt % Nafion solution (product number: 274704) was purchased from Sigma-Aldrich, Japan K.K. (Tokyo, Japan), and its cation was exchanged by the addition of a 0.1 M KOH aqueous solution. Multiwall carbon nanotubes (CNTs, 90%) were purchased from Nanocyl SA. (Sambreville, Belgium) and functionalized as reported in our previous paper.^41^ Iridium oxide ((IrO_2_), 75% Ir, product number: TEC77100) was purchased from Tanaka Kikinzoku Kogyo (Tokyo, Japan). One M KOH solution (volumetric analysis grade, Wako Pure Chemical Industries, Ltd. (Osaka, Japan)) was used as the electrolyte for the in situ XAFS measurements as received. All other chemicals were purchased from Wako Pure Chemical and used as received.

### Catalyst Synthesis

The Mn-based nanospinels were synthesized via an alcohol reduction process. The synthesis of Li–, Mg–, Zn–, Cu–, and Co–Mn nanospinels was carried out according to the literature with modifications.^35−37,42^ For Ni–Mn nanospinels, Bu_4_NMnO_4_ powder (2 mmol) was slowly added into the NiCl_2_·6H_2_O (2 mmol) solution in methanol and ethylene glycol dimethyl ether (50 mL, 1/1 by volume) under vigorous stirring, followed by stirring for 1 h. The reaction solution was filtered, washed with water and ethanol several times, and dried to yield the NiMn_2_O_4_ nanospinel.

NiMn_2_O_4_ and CoMn_2_O_4_ normal spinels were synthesized via a modified sol–gel process with reference to our previous paper.^43^ Ni(NO_3_)_2_ (2.5 mmol), Mn(NO_3_)_2_ (5.0 mmol), and citric acid (15.0 mmol) were dissolved in 5 mL of deionized water in a crucible, followed by the addition of 5 mL of ethylene glycol. The solution was heated at 70 °C for 24 h, and the resulting gel was calcined at 200 °C for 12 h and then at 800 °C for 10 h to yield 0.415 g of the NiMn_2_O_4_ normal spinel as a black powder. The CoMn_2_O_4_ normal spinel was synthesized by replacing Ni(NO_3_)_2_ to Co(NO_3_)_2_.

### Material Characterizations

X-ray diffraction (XRD) patterns were collected using a D2 PHASER XE-T Edition. The Rietveld refinement was performed using the RIETAN-FP program.^44^ Scanning electron microscopy (SEM) energy-dispersive X-ray (EDX) elemental analysis was performed using a JSM-IT510A scanning electron microscope. Transmission electron microscopy (TEM) images and selected area electron diffraction (SAED) patterns were obtained using an EM-002B transmission electron microscope. For characterizations of the NiMn_2_O_4_ normal spinel, SEM and TEM images were acquired using HITACHI S-5500 and H-7650 instruments operated at 5.0 and 100 kV, respectively. Brunauer–Emmett–Teller (BET) surface area analysis with N_2_ adsorbents was carried out using a BELSORP MAX G surface area and pore size distribution analyzer. Raman spectroscopy was performed using a confocal Raman microscope (LabRAM HR Evolution, HORIBA Ltd.) with a 532 nm excitation laser. The spectra were recorded with a resolution of 0.2 cm^–1^ using a 600 gr/mm grating at ambient temperature.

### Electrochemical Measurements

The electrocatalytic OER activities of catalysts were evaluated on a potentiostat HZ-7000 (HOKUTO DENKO Corp., Tokyo, Japan) equipped with an electrode rotator (HR-301, HOKUTO DENKO Corp.) and a frequency response analyzer (HZA-FRA1) using a rotating disk electrode (RDE) with a disk area of 0.196 cm^2^ made of glassy carbon as the working electrode. Figure S1a shows the electrolytic cell for the OER analysis. A Pt wire and Hg/HgO (1 M KOH) were used as the counter and reference electrodes, respectively. The electrolytic cell is equipped with a gas inlet to saturate the electrolyte solutions with N_2_ (30 min) or O_2_ (1 h) bubbling prior to measurements. The potential on the Hg/HgO electrode was converted to a reversible hydrogen electrode (RHE). The calibration of the reference electrode was carried out using the following equation (eq 1) as reported by Li et al.^45^
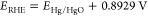
1The working electrodes for the measurements were prepared as follows. The catalyst slurries were fabricated by mixing 1.5 mg of catalyst powder, 3.5 mg of functionalized CNTs, 0.144 mL of 3.3 wt % K^+^ ion-exchanged Nafion, 1.2 mL of ethanol, and 1.2 mL of water, homogenizing for 3 min at 4 °C and sonicating for 2 h. Then, 10 μL of the slurry was dropped onto the RDE and dried by rotation at 600 rpm in air. The catalyst loading amount was set to 0.030 mg cm_disk_^–2^. The IrO_2_ electrode was also fabricated by the same protocol.

The working electrodes were pretreated by five cyclic voltammetry (CV) scans between 0.1 and 1.2 V vs RHE at a scan rate of 50 mV s^–1^ in N_2_-saturated 1 M KOH without disk rotation. The OER activities of the catalysts were evaluated by CV scan sweeps between 1.2 and 1.8 V vs RHE with a rotation speed of 1600 rpm at a scan rate of 10 mV s^–1^ in O_2_-saturated 1 M KOH at ambient temperature. To ensure the theoretical equilibrium potential of the OER for 1.23 V vs RHE, the electrolyte solutions were saturated with O_2_.^46^ The OER polarization curves were recorded as the average currents of forward and backward sweeps of the 10th CV cycle to cancel capacitive currents.^14^ Electrolyte solutions were saturated by O_2_ bubbling for 1 h before measurements. The *iR* losses were compensated using the following equation (eq 2) with the measured current (*i*) and electrolyte resistance (*R*), which were determined by the ac impedance
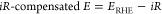
2Electrochemical impedance spectroscopy (EIS) to examine the charge-transfer resistance (*R*_ct_) through the interfaces between the nanospinel catalysts on RDEs and the electrolyte KOH solutions was performed in a frequency range from 100 kHz to 0.1 Hz at 1.65 V with an amplitude of 5 mV after the OER analysis. The collected Nyquist plots were fitted based on a basic equivalent circuit, comprising the solution resistance (*R*_s_), *R*_ct_, and constant-phase element (CPE), as illustrated in Figure S2, to estimate the *R*_ct_ using EIS software (version 1.2.2, HOKUTO DENKO Corp.).

### Operando X-ray Absorption Fine Structure Measurements

The operando XAFS measurements for the NiMn_2_O_4_ nanospinel were carried out at the BL11S2 beamline in the Aichi Synchrotron Radiation Center (Seto, Japan) using an in-house designed PEEK cell setup, as shown in Figure S1b. Using a seven-element silicon drift detector, the spectra were collected in fluorescence mode for the Mn and Ni K-edges. Mn and Ni foils were measured for energy calibrations. The aforementioned catalyst slurry of NiMn_2_O_4_ was dropped onto a conductive graphite sheet (EYGS182307, Panasonic Industry Co., Ltd., Kadoma, Japan) to prepare the working electrodes. A platinum foil and Hg/HgO (1 M NaOH) were used as counter and reference electrodes, respectively. O_2_ gas was flowed during the measurements. The potentials in the OER region were applied using the potentiostat HZ-7000, and the operando XAFS spectra were recorded at the open-circuit potential and the desired potential. The electrode was equilibrated at each potential for 30 s before measurements. The EXAFS spectral analysis and the fitting of the normalized EXAFS spectra were then performed by Demeter Athena software and Demeter Artemis software, respectively.^47^ The fitting of the EXAFS spectra was performed using a *k*^3^-weighting and a Hanning window from *k* = 3–9 Å for the Ni K-edge and *k* = 3–11 Å for the Mn K-edge, respectively. The fitting was conducted by R-space with a *R*-range of 1.0–4.0 Å. For the first scan at 1.4 V of the Mn K-edge, the Debye–Waller factor became negative. Therefore, the restraint was applied for obtaining a positive Debye–Waller factor.

## Results and Discussion

### Synthesis and Characterizations of Catalysts

**Figure 1:**
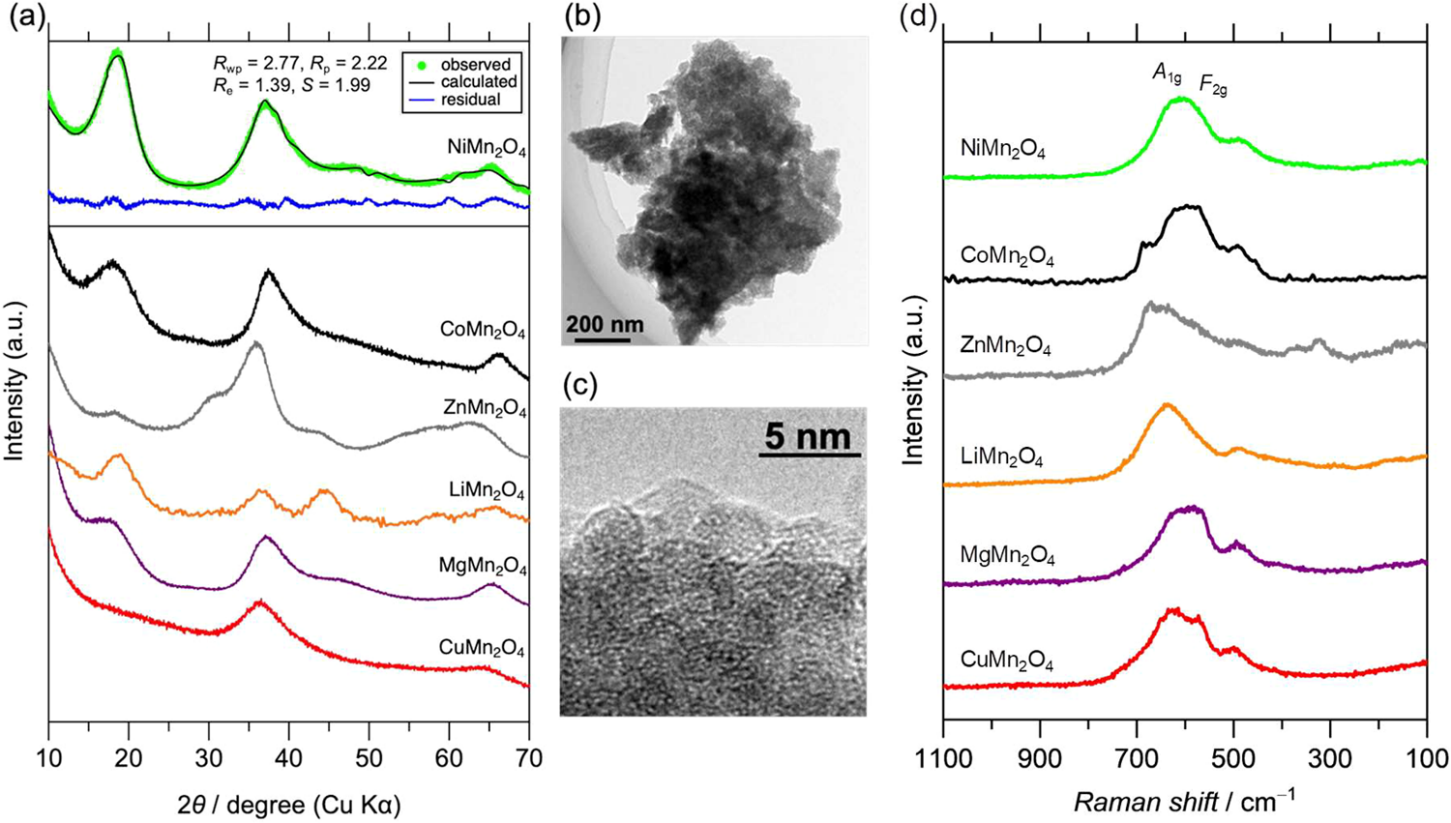
(a) XRD patterns of the nanospinels. (b, c) TEM and HR-TEM images of NiMn_2_O_4_, respectively. (d) Raman spectra of the nanospinels.

The Mn-based spinels with a nanometer size (nanospinels) were synthesized via an alcohol reduction method. XRD patterns (Figure 1a) show broad peaks due to their small crystallite size; patterns of Co–, Zn–, Li–, Mg–, and Cu–Mn nanospinels match the previous literature.^35−37,42^ The Ni–Mn nanospinel, newly synthesized, is attributed to the cubic NiMn_2_O_4_ typical spinel from the Rietveld refinement, except for Zn–Mn nanospinels that are a tetragonal spinel structure. SEM-EDX supports the formula by analyzing the elemental ratio of Ni/Mn = 1/2. Figure 1b,c shows TEM images. The observed particles were attributed to NiMn_2_O_4_ from the SAED patterns (Figure S3). The NiMn_2_O_4_ nanospinel forms large secondary particles of ca. 1 μm; on the other hand, the primary particles are about 5 nm in size, implying the successful formation of ultrasmall particles via the room-temperature wet process. The BET surface area of 342 m^2^ g^–1^ also supports the ultrasmall primary particles. In addition, Co–, Zn–, Li–, Mg–, and Cu–Mn nanospinels exhibited the BET surface areas of 284, 234, 371, 436, and 295 m^2^ g^–1^, respectively.^36,37,42^ The BET surface areas were used to normalize their OER current density for determining their OER specific activities in the next following sections. Furthermore, the electronic states for the synthesized Mn-based nanospinels were examined by XAFS measurements. Figure S4 shows the Mn K-edge X-ray absorption near-edge structure (XANES) spectra. The valence state of Mn was evaluated based on the energy at N.A. = 0.7, which is known to reflect the valence state as reported previously.^37^ This result indicates that valence states of Mn in the nanospinels follow the order: Zn– < Cu– < Ni– ∼ Co– ∼ Li– ∼ Mg–Mn nanospinels.^35−37,42^Figure 1d depicts the Raman spectra of the nanospinels. The spectra demonstrated broad peaks around 620 and 570 cm^–1^ that can be assigned as the symmetrical Mn–O stretching vibration in octahedral sites (*A*_1g_ and *F*_2g_, respectively).^48^ The characteristics agreed to the reported nanospinel with a cubic spinel structure.^35^ On the other hand, the ZnMn_2_O_4_ nanospinel has a tetragonal structure and thus did not exhibit a clear *F*_2g_ signal.

Furthermore, NiMn_2_O_4_ and CoMn_2_O_4_ spinel oxides were also synthesized via the sol–gel process (normal spinel) as the control samples. Their powder XRD patterns were consistent with that for the previously reported cubic NiMn_2_O_4_ spinel [space group: *Fd*-*3m*, ICSD: 174001] and the tetragonal CoMn_2_O_4_ spinel [space group: *I41*/*amd*, JCPDS: 007-9472] (Figures S5 and S8). Figure S6 shows the SEM image of the NiMn_2_O_4_ normal spinel with 300–600 nm-sized spherical particles, which were 2 orders of magnitude larger than those of the above-mentioned Mn-based nanospinels. Figure S7 depicts the TEM image of the NiMn_2_O_4_ normal spinel. The TEM image shows that the primary particle size of the NiMn_2_O_4_ normal spinel is also several hundred nanometers, which is well consistent with the SEM image. Additionally, its BET specific surface area was 2 m^2^ g^–1^, which was much smaller than those of the nanospinels. On the other hand, Figure S9 shows the SEM image of the CoMn_2_O_4_ normal spinel with 400–1000 nm-sized spherical particles, which were also 2 orders of magnitude larger than those of the above-mentioned Mn-based nanospinels. The TEM image of CoMn_2_O_4_ shown in Figure S10 is also very consistent with the SEM image. Its BET specific surface area was 1 m^2^ g^–1^, which was close to that of the NiMn_2_O_4_ normal spinel. The Raman spectra of the NiMn_2_O_4_ and CoMn_2_O_4_ normal spinels are shown in Figure S11. These spectra were consistent with the reported characteristic Raman shifts for these compounds.^49^ In comparison with these normal spinels, the peaks of the NiMn_2_O_4_ and CoMn_2_O_4_ nanospinels were broadened, as exhibited in Figure S11. This can be attributed to the large fraction of the surface due to their ultrasmall 5 nm-sized particles.

### Electrocatalytic OER Performances

**Figure 2:**
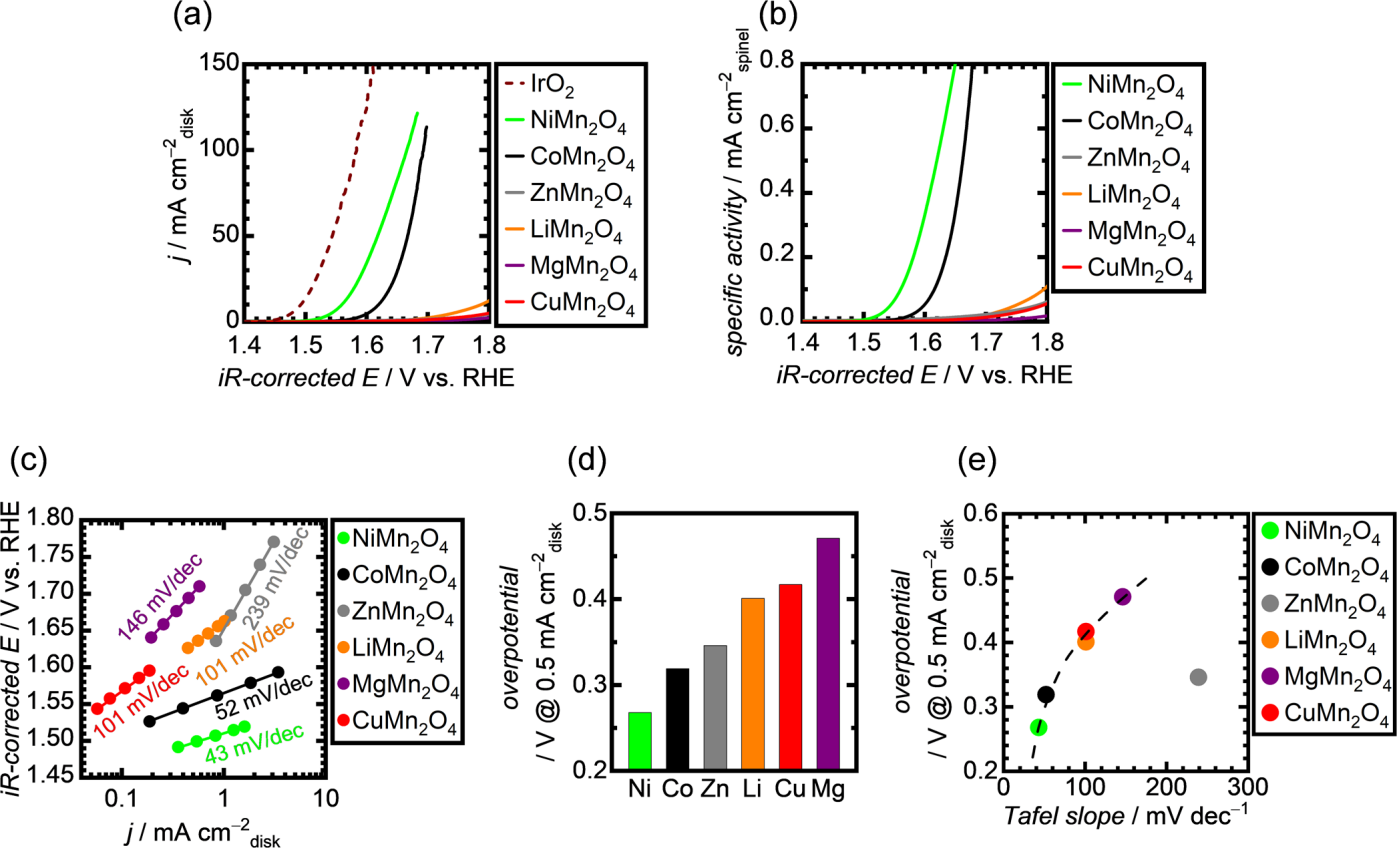
OER performances of the Mn-based nanospinels. (a) Polarization curves in O_2_-saturated 1 M KOH with a rotation speed of 1600 rpm. (b) OER specific activities, which were obtained by normalizing the current densities of polarization curves in panel (a) using BET surface areas of the nanospinels. (c) Measured Tafel plots. (d) Comparison of OER overpotentials at 0.5 mA cm_disk_^–2^. (e) OER overpotentials as a function of the Tafel slopes.

First, the electrochemical redox characteristics of the nanospinels were checked by CV measurements. The CV curves of the nanospinels shown in Figure S12 depict that the shapes of Mn redox peaks are different among the nanospinels, which indicates that the nanospinels had different surface characteristics. In addition, a large Mn reduction peak was detected at 0.7–0.8 V for the NiMn_2_O_4_ nanospinel, but other nanospinels did not exhibit it. This peak can be assigned to Ni reduction, and thus, the NiMn_2_O_4_ nanospinel has different surface environments of Mn species from other nanospinels. The electrocatalytic OER performances of the synthesized Mn-based nanospinels were evaluated using O_2_-saturated 1 M KOH. Figure 2a shows the *iR*-compensated OER polarization curves for the nanospinels with the benchmark IrO_2_, indicating higher OER currents for NiMn_2_O_4_ and CoMn_2_O_4_ than those of ZnMn_2_O_4_, LiMn_2_O_4_, CuMn_2_O_4_, and MgMn_2_O_4_. To eliminate the effects of the particle size, i.e., the surface area of the catalyst particles on the OER activity, the recorded OER currents were normalized using each BET surface area of the catalyst particles. Consequently, the *iR-*compensated polarization curves based on the OER specific activity are shown in Figure 2b. The results found the order of their intrinsic OER activity to be NiMn_2_O_4_ > CoMn_2_O_4_ > others. In addition, Tafel plots shown in Figure 2c demonstrated similar trends, where NiMn_2_O_4_ and CoMn_2_O_4_ had Tafel slopes smaller than those of the other nanospinels, indicating better OER kinetics. The polarization curve of each nanospinel was also used to determine the onset OER overpotential, which is defined as the difference between the theoretical OER (1.23 V vs RHE) and recorded potentials, when reaching a current density of 0.5 mA cm_disk_^–2^. Generally, the OER overpotential is defined on the basis of potentials with a current density of 10 mA cm_disk_^–2^, but low-active catalysts in this study did not reach this current density. Thus, the OER overpotentials of the catalysts were compared at 0.5 mA cm_disk_^–2^. NiMn_2_O_4_ showed an overpotential of 0.27 V, and this value was lower than those of the other nanospinels, as shown in Figure 2d. The overpotentials of the nanospinels follow the order: Ni < Co < Zn < Li < Cu < Mg. To further discuss the OER catalysis on the nanospinels, their overpotentials were plotted against their Tafel slopes, as displayed in Figure 2e, which shows an excellent correlation. The smaller the Tafel slope, the lower is the overpotential, indicating a more efficient OER activity. However, ZnMn_2_O_4_ deviates from the other distributions, exhibiting a large Tafel slope but a relatively low overpotential. This deviation may be attributed to the unique tetragonal structure of ZnMn_2_O_4_, as opposed to the cubic structures of the other nanospinels. The distinct structure of ZnMn_2_O_4_ is further supported by the Raman spectrum displayed in Figure 2d, which shows a diminished *F*_2g_ signal. It is interesting that the trend of A-site-dependent OER activity among these nanospinels, i.e., NiMn_2_O_4_ > CoMn_2_O_4_ > ZnMn_2_O_4_ > LiMn_2_O_4_ > CuMn_2_O_4_ > MgMn_2_O_4_, is different from the reported one for the normal spinel (CoFe_2_O_4_ > CuFe_2_O_4_ > NiFe_2_O_4_).^38^

**Figure 3:**
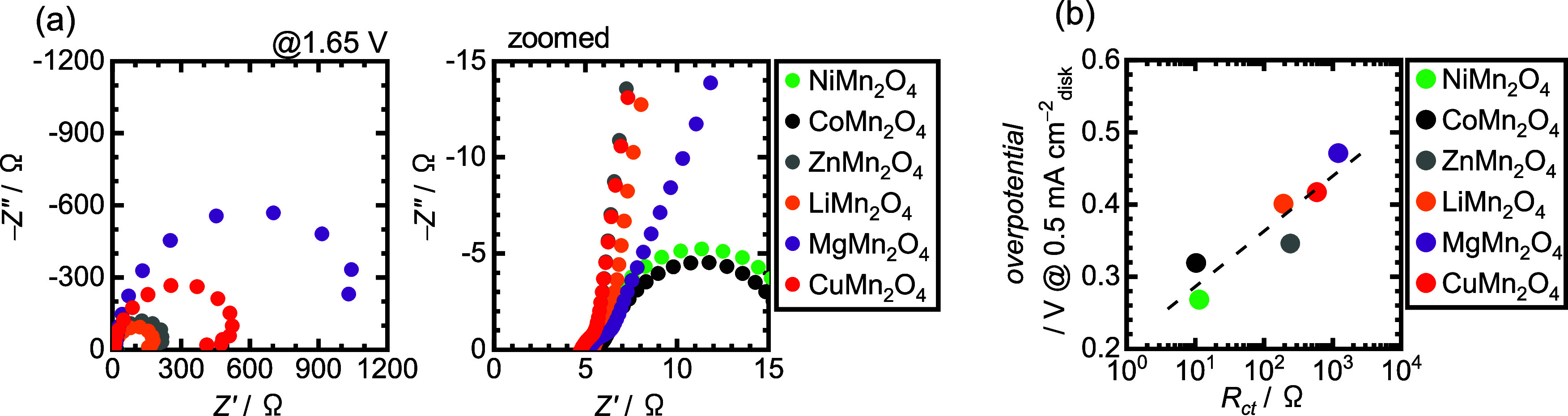
(a) Nyquist plots from EIS recorded from 100 kHz to 0.1 Hz at 1.65 V vs RHE in O_2_-saturated 1 M KOH. (b) OER overpotential as a function of *R*_ct_.

Electrochemical impedance spectroscopy (EIS) was also carried out to examine the electrochemical kinetics of the OER process on the nanospinels. Figure 3a displays the measured Nyquist plots from EIS at 1.65 V vs RHE, and they were fitted using a basic equivalent circuit, as illustrated in Figure S2, to estimate the charge-transfer resistance (*R*_ct_) values. *R*_ct_ represents the Faradaic charge transfer across the electrode/solution interface, and the Nyquist plots should be measured at the potential of the OER region to analyze the interfacial charge transfers for the OER process.^50^ Thus, EIS measurements were performed at 1.65 V. Figure 3b shows the plots of overpotentials of each nanospinel vs *R*_ct_. The *R*_ct_ values of the nanospinels displayed a correlation with the OER overpotentials, and the plots exhibited a linear relationship because the plot of the logarithmic Faradaic resistance against *E* is equal to the Tafel slope.^51^ The results indicated the good consistency of EIS results with the OER polarization curves, and NiMn_2_O_4_ and CoMn_2_O_4_ had better OER kinetics than those of the other nanospinels.

**Figure 4:**
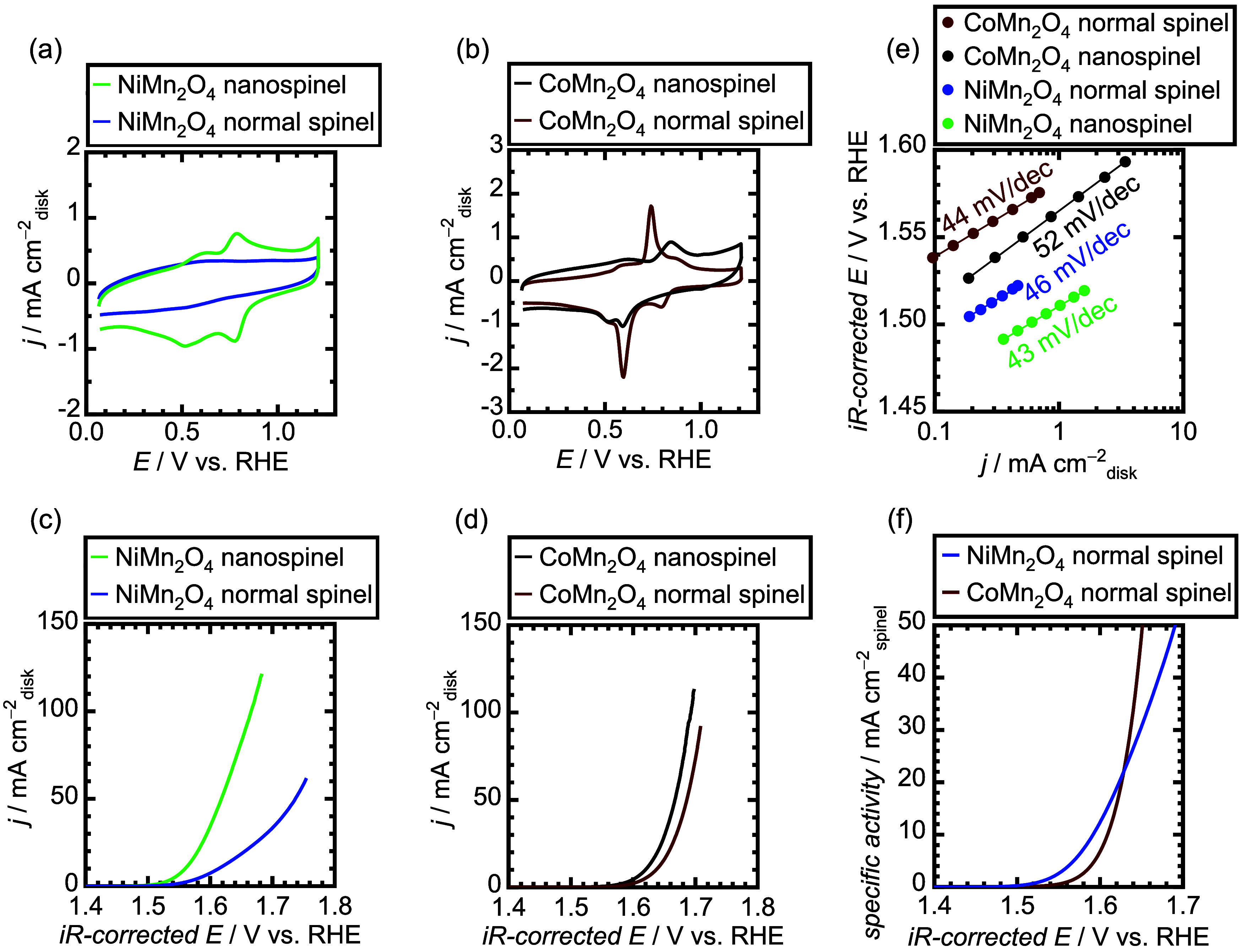
Comparison of electrochemical measurements for NiMn_2_O_4_ and CoMn_2_O_4_ nanospinels with normal spinels. (a, b) CV measurements for NiMn_2_O_4_ and CoMn_2_O_4_, respectively, in N_2_-saturated 1 M KOH without rotation. (c, d) Polarization curves for NiMn_2_O_4_ and CoMn_2_O_4_, respectively, in O_2_-saturated 1 M KOH with a rotation speed of 1600 rpm. (e) Comparison of Tafel slopes for NiMn_2_O_4_ and CoMn_2_O_4_ nanospinels with normal spinels. (f) OER specific activities of NiMn_2_O_4_ and CoMn_2_O_4_ normal spinels in O_2_-saturated 1 M KOH with a rotation speed of 1600 rpm.

To prove the merits of using nanospinels, we compare the NiMn_2_O_4_ nanospinel with the NiMn_2_O_4_ normal spinel, which has a much lower BET specific surface area (2 m^2^ g^–1^). Figure 4a displays CV curves during pretreatments before OER measurements for the NiMn_2_O_4_ nanospinel and the NiMn_2_O_4_ normal spinel in a lower potential region than the theoretical OER potential. The NiMn_2_O_4_ nanospinel exhibits clear redox peaks of Ni and/or Mn between 0.5 and 0.9 V, while the CV curve for the NiMn_2_O_4_ normal spinel has much smaller peaks despite their same crystal structures. This indicates that the NiMn_2_O_4_ nanospinel possesses a much larger electrochemical active surface area than the NiMn_2_O_4_ normal spinel. Thus, Figure 4c clearly shows that the NiMn_2_O_4_ nanospinel has a much better OER activity than the NiMn_2_O_4_ normal spinel, namely, the NiMn_2_O_4_ nanospinel exhibited a lower OER overpotential by 0.026 V @ 0.5 mA cm_disk_^–2^ and 0.058 V @ 10 mA cm_disk_^–2^ than the normal spinel. The improvement of the OER performance corroborated the merit of nanospinels as electrode materials toward the electrocatalytic OER process. Furthermore, the CoMn_2_O_4_ nanospinel was compared with the CoMn_2_O_4_ normal spinel with a BET specific surface area of 1 m^2^ g^–1^. Interestingly, the CoMn_2_O_4_ normal spinel possesses significantly high redox peaks in its CV curve, as shown in Figure 4b, and the CoMn_2_O_4_ nanospinel displays only a slight increase in the level of the OER activity compared with the CoMn_2_O_4_ normal spinel, as presented in Figure 4d. The Tafel slopes for the nanospinels and normal spinels exhibited similar values, as shown in Figure 4e. To further discuss the characteristics of normal spinels, their OER specific activities were compared and are shown in Figure 4f. The NiMn_2_O_4_ normal spinel exhibited a lower OER onset potential than the CoMn_2_O_4_ normal spinel, which was consistent with the nanospinels shown in Figure 2b. Contrarily, at the high-current regions, the CoMn_2_O_4_ normal spinel displays a better OER specific activity than the NiMn_2_O_4_ normal spinel. Because the OER current for the NiMn_2_O_4_ normal spinel was raised at a lower potential, the NiMn_2_O_4_ normal spinel possessed a higher intrinsic OER activity. In contrast, the larger current of the CoMn_2_O_4_ normal spinel at a high potential might be due to the favorable charge transfer within the crystal. In cases of normal spinels that are synthesized via the sol–gel method, NiMn_2_O_4_ and CoMn_2_O_4_ normal spinels bear cubic and tetragonal crystal systems, respectively. Previous literature investigated cubic MnCo_2_O_4_ and tetragonal CoMn_2_O_4_ and found that the latter has a greater charge difference between metal sites, enhancing charge mobility within the bulk crystal of the compound.^52^ The facilitated charge transfer is beneficial for electrochemical OER process,^53−56^ which can lead to greater currents at a higher potential. The favorable charge transfer in the tetragonal CoMn_2_O_4_ normal spinel is also supported by the large redox peak in its CV curve, as shown in Figure 4b. The trend between NiMn_2_O_4_ and CoMn_2_O_4_ normal spinels is opposite to that of the nanospinels shown in Figure 2b. It is noteworthy that the crystal system of the CoMn_2_O_4_ nanospinel is cubic and the same as that of the NiMn_2_O_4_ nanospinel due to the synthetic process for nanospinels, the alcohol reduction method. Consequently, the trend of the OER performance of the nanospinel is different from that of the normal spinels, and the NiMn_2_O_4_ nanospinel possess a better OER performance compared with the CoMn_2_O_4_ nanospinel.

When comparing the NiMn_2_O_4_ nanospinel with the previously reported spinel-type electrocatalysts bearing 3*d* transition metals, the OER performance of the NiMn_2_O_4_ nanospinel was one of the best OER electrocatalysts, as shown in Table S1, even though the OER activity was lower than that of the benchmark IrO_2_ (Figure 2a). Thus, the OER evaluation demonstrated that NiMn_2_O_4_ is the most active OER electrocatalyst among the Mn-based nanospinels. To execute the long-term durability test for NiMn_2_O_4_, repeated potential cycles were carried out. Figure S13a demonstrates that 1000 repeated potential cycles induced a gradual current loss. The Raman spectrum after 1000 cycles, as depicted in Figure S13b, shows that the Raman peak has shifted to the lower wavenumber region, and a new peak has appeared at 900 cm^–1^. Because similar spectral changes for NiMn_2_O_4_ were also detected after only initial measurements (see below), it is speculated that not only changes in the catalyst but also the corrosion degradation of the CNT^57^ were the main reasons for the decrease in the current during the repeated potential cycles.

### Insights into the Reaction Mechanism of the OER for the NiMn_2_O_4_ Nanospinel

To investigate the electronic states of metal active centers, the in situ X-ray absorption fine structure (XAFS) analyses of NiMn_2_O_4_, the best OER catalysts among the synthesized samples, was conducted. First, we checked the consistency of the electrochemical measurement between the typical RDE setup (Figure S1a) and the in-house cell setup for operando XAFS measurements (Figure S1b). Figure S14a,b displays the CV curves for pretreatment and the OER polarization curves of the NiMn_2_O_4_ nanospinel, respectively, which were obtained using the RDE and the in-house cell setups. The two redox peaks were observed for NiMn_2_O_4_ both for the typical RDE setup and the in-house cell for operando XAFS measurements, and CV results are essentially similar. These results confirmed the consistency between the two cell setups. Although the commercially available carbon papers were used as the working electrode, the CV curve is essentially same as the ones measured using GC electrodes. In addition, the OER polarization curves are also essentially same as the one measured by RDE measurement. These results indicate that our house-made operando XAFS cell is suitable to analyze the reaction mechanism of the OER by XAFS measurement.

**Figure 5:**
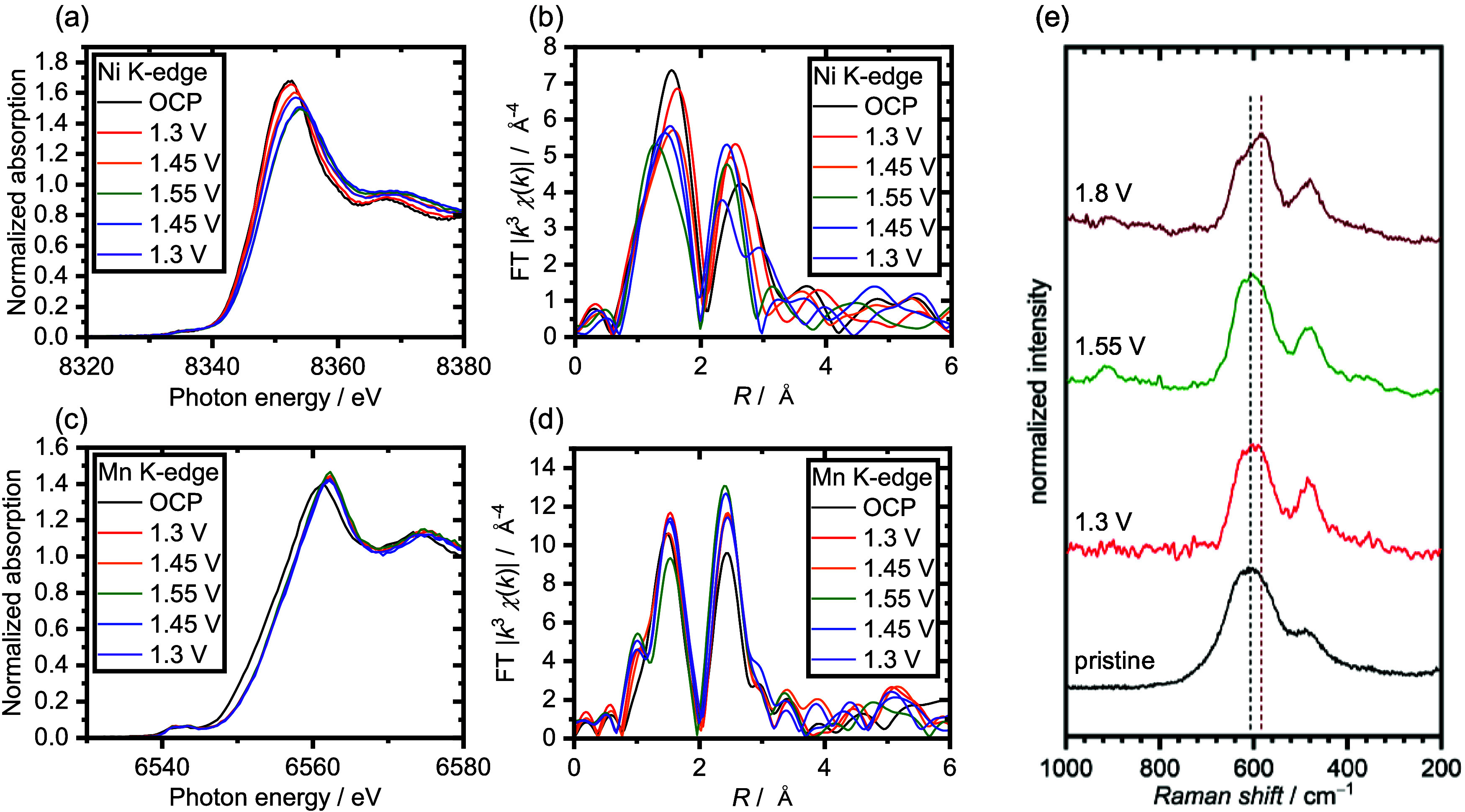
(a, c) Ni and Mn K-edge spectra from XANES analysis, respectively. (b, d) Fourier-transformed Ni and Mn K-edge spectra from EXAFS analysis, respectively. (e) Raman spectra of NiMn_2_O_4_ nanospinels after application of each potential.

Figure 5 shows the X-ray absorption near-edge structure (XANES) and the Fourier transform extended X-ray absorption fine structure (FT-EXAFS) spectra of Ni (Figure 5a,c) and Mn (Figure 5b,d) K-edges, respectively. This study conducted the operando XAS analysis by changing the potential range to 1.3–1.55 V vs RHE. The XANES spectrum of the Ni K-edge shifted to the higher energy region as the applied potential increased (Figure 5a). This result indicates that a valence-state change of Ni occurred, and Ni species are oxidized possibly from Ni^2+^ to Ni^3+^/Ni^4+^, as reported previously.^58^ The Ni K-edge XANES spectra shifted to a lower energy region as the applied potential decreased. However, it did not completely revert to the spectra observed at the open-circuit potential. Notably, the XANES spectrum was not completely reversible in the repeated cycle experiments (Figures S15a and S16a). These results indicate that the coordination structure or electronic structure of Ni changes during the first cycle, whereas the redox behavior of Ni is reversible in the potential range for this measurement after second cycles. On the other hand, for Mn K-edge, no essential change was observed in the XANES spectra in the applied potential region (Figures 5c and S16b). This result suggests that either of the redox peaks observed in the CV shown in Figure S14a is Mn^3+^/Mn^4+^, and no Mn redox occurs in the OER potential region.

For a detailed analysis, we also investigated the shifts of the Ni and Mn K-edges by plotting the midpoint of the XANES spectra (Figure S16). Regarding the Ni K-edge (Figure S16a), the spectra gradually shifted to the higher energy region as the applied potential increased. However, the peak shift was not completely reversible during repeated cycle tests. This result indicates that the electronic states of Ni are only partially reversible. In contrast, the Mn K-edge did not show significant changes (Figure S16b). These results also suggest that the electronic-state changes of Ni species occur during the potential sweep and could be responsible for the OER.

Next, Figure 5b,d shows the Ni and Mn K-edge FT-EXAFS spectra, respectively. For the Ni K-edge (Figure 5b), the peaks corresponding to the first coordination sphere, mainly assignable to Ni–O, slightly shifted to shorter distances and got smaller intensities at higher potential regions for the first potential sweep. For repeated measurement of Ni–K-edge EXAFS spectra (Figure S15b), the bond distance (*x*-axis) of the Ni–O bond around 1.6 Å changed following the applied potential, with a longer bond length in the higher potential region, but the intensity did not change after the first scan. The second coordination sphere around 2.4–2.6 Å usually reflects the bond of Ni–O–Ni for spinel oxides. The bond length slightly decreased in the first potential sweep to the higher potential region. The peak intensities at the second coordination sphere got stronger in the higher potential region and smaller in the lower potential region for the second cycle. This result suggests that the coordination structure change is reversible after the second potential scan. The peak position reflects the Ni atom sites in spinel oxides. It is reported that the metal-O-metal bond length and metal–metal distance corresponding to tetrahedral 8a sites are longer than those corresponding to 16d octahedral sites.^20,59^ In this research, peaks around 2.4 Å show the Ni atoms at the octahedral site, and the peak around 2.6 Å shows the Ni atoms at the tetrahedral site. Based on these results, it is suggested that Ni’s coordination structure changed from a tetrahedral structure (8a sites of spinel oxides) to an octahedral structure (16d sites of spinel oxides or hydroxide structure). For Mn K-edge FT-EXAFS, the peak intensity in the first coordination region became weaker as the potential increased. In contrast, the peak intensity in the second coordination region became stronger as the potential swept toward higher potentials, although no significant peak shift was observed (Figure 5d), which means no site occupancy change occurs for Mn atoms.

In addition, the fitting of in situ EXAFS for Ni and Mn K-edges was performed to examine the structural analysis of NiMn_2_O_4_ during the OER and the result is listed in Tables S2 and S3. The representative fitting results are shown in Figure S17. Figure S18 shows the changes in M–O and M–M bond distances as a function of the applied potential for both the Ni K-edge and the Mn K-edge. At the Ni K-edge (Figure S18a), the first shell (Ni–O) shows a clear decrease in the bond distance with the increasing potential. The second shell (Ni–M) also gradually decreased as the applied potential increased higher than 1.4 V. A detailed analysis suggests that the shortening of the Ni–O and Ni-M bond distances under higher potential regions could be attributed to the oxidation of Ni ions.^60^ Interestingly, distinct changes in Ni–M bond distances were observed for the first, second, and third scans at 1.3 V. In previous studies, it is reported that the structure change of spinel oxide to hydroxide formation occurs under OER conditions;^20,59^ however, the CN value for Ni–O and Ni-metal (Ni-M) bonds is smaller compared with the hydroxide structure. Therefore, we hypothesize that this CN value change in Ni species is not originated by the single factor but is likely the result of multiple factors, including surface reconstruction involving a phase transition to hydroxides, variations in the occupancy of metal cation sites under the applied potential,^61^ and the introduction of oxygen vacancies due to the ultrasmall nanoparticle size. In contrast, at the Mn K-edge (Figure S18b), the Mn–O bond and the Mn–M distance remain relatively similar, regardless of the potential application, suggesting a limited effect of the potential applications to Mn species. If the degree of inversion between Ni and Mn species changes, the Mn–M bond length increases because M–M bonds in tetrahedral sites are longer than those in octahedral sites. However, the fitting results for the Mn–M bond did not show a significant increase, as shown in Figure S18b. Therefore, it is suggested that the origin of the structural change is primarily the formation of hydroxides. This result implies that Ni species play an important role in the OER, and Mn species mainly function as the host of active centers composed of Ni species. Furthermore, Figure 5e depicts the Raman spectra for NiMn_2_O_4_ after application of each potential, 1.3, 1.55, and 1.8 V. The peak of the main band was shifted to the lower wavenumber region in the case of application of a higher potential, which also proved the electrochemical transformation of NiMn_2_O_4_ at higher potentials. In previous reports, the surface of the metal or metal oxide catalysts could be transformed into the hydroxide species during the potential applications.^62,63^ Taken together, this study indicates that the structure of NiMn_2_O_4_ transformed into a hydroxide structure, which is similar to Mn–Ni hydroxides. The previous paper^64^ presented that Mn–Ni hydroxides on the surface of NiMn oxide led to an excellent OER performance. Therefore, favorable surface reconstruction of the OER took place on the surface of the NiMn_2_O_4_ nanospinel. Hence, the nanospinels are promising compounds as OER electrocatalysts, and this study can pave a new way for the innovation of hydrogen production via electrochemical alkaline water splitting.

## Conclusions

This work systematically investigated the influence of the A-site metals in a Mn-based nanospinel (AMn_2_O_4_) on their electrocatalytic OER activity. The series of the Mn-based nanospinels with A = Li, Mg, Zn, Ni, Cu, or Co were synthesized via similar processes, and their OER activities were evaluated. The NiMn_2_O_4_ nanospinel exhibited the highest OER activity among the Mn-based nanospinels. Furthermore, the OER activity on the NiMn_2_O_4_ nanospinel was dramatically enhanced compared with the NiMn_2_O_4_ normal spinel with a much larger particle size, and the result proved the advantage of nanospinels as an OER electrocatalyst. Moreover, the OER performance of the NiMn_2_O_4_ nanospinel made it one of the best electrocatalysts among the previously reported bimetal spinel oxides. Finally, we performed operando XAFS measurements using in-house electrochemical cells and demonstrated that the surface of the NiMn_2_O_4_ nanospinel was electrochemically transformed to Mn–Ni hydroxide that is preferable for the OER process. This work uncovered a good potential of nanospinels as an OER electrocatalyst and provided a guideline for the selection of metal components in the nanospinel structure.

## Supporting Information Available

Supplementary figures and tables, additional experimental data, computational details, and comparison of OER activity with previously reported catalysts (PDF)
