# Supplementary material for: Ultrasmall Manganese Nanospinels Produced via an Alcohol Reduction Method and Their Electrocatalytic Oxygen Evolution Reactivity
Source: ACS Appl Mater Interfaces. 2025 Apr 7;17(15):22487–97. doi: 10.1021/acsami.4c18777 (PMC12012771; doi:10.1021/acsami.4c18777)
Supplement: Supplementary file 1 — am4c18777_si_001.pdf [file am4c18777_si_001.pdf]

Supporting Information

Ultrasmall Manganese Nanospinels produced via an Alcohol Reduction Method and their Electrocatalytic Oxygen Evolution Reactivity

Yuuki Sugawara,*[†#] Kazuyuki Iwase,*[‡#] Reona Iimura,[‡] Takashi Yabu,[$] Akira Nasu,[$] Masaki Matsui,[$] Itaru Honma,[‡] Takeo Yamaguchi,[†] Hiroaki Kobayashi*[$]

[†]*Laboratory for Chemistry and Life Science, Institute of Integrated Research, Institute of Science Tokyo, R1-17, 4259 Nagatsuta-cho, Midori-ku, Yokohama, Kanagawa 226-8501, Japan*

[‡]*Institute of Multidisciplinary Research for Advanced Materials, Tohoku University, 2-1-1 Katahira, Aoba-ku, Sendai, Miyagi 980-8577, Japan*

[§]*Department of Chemistry, Faculty of Science, Hokkai-do University, Kita 10, Nishi 8, Kita-ku, Sapporo, Hokkaido 060-0810, Japan*

*Corresponding author E-mail: h.kobayashi@sci.hokudai.ac.jp; kazuyuki.iwase.a6@tohoku.ac.jp; sugawara.y.aa@m.titech.ac.jp.

#: Y.S. and K.I. contributed equally to this work.

Supplementary Figures and Tables

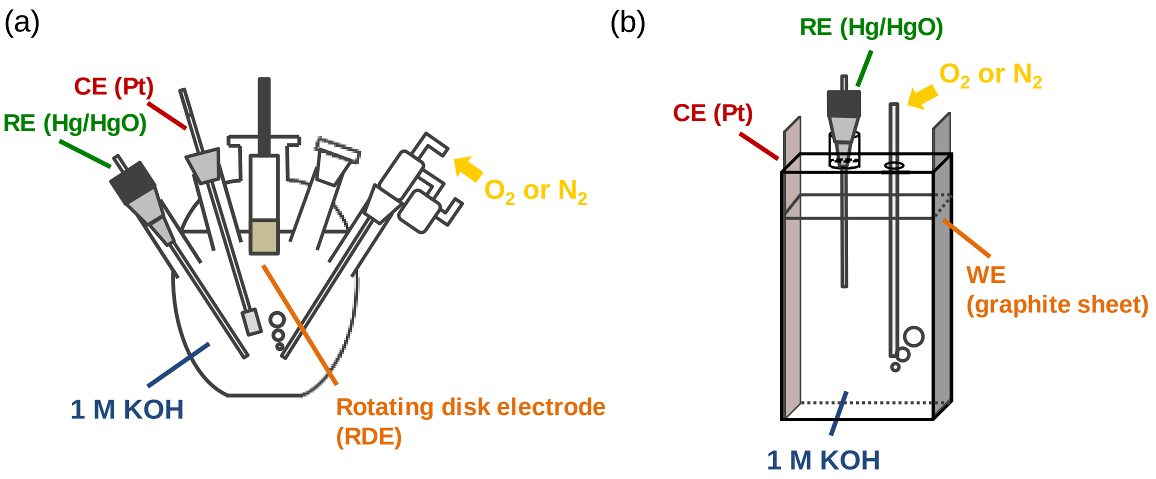

Figure S1. A set-up for electrochemical measurements carried out using (a) RDE and (b) PEEK cell for *operando* XAFS measurements.

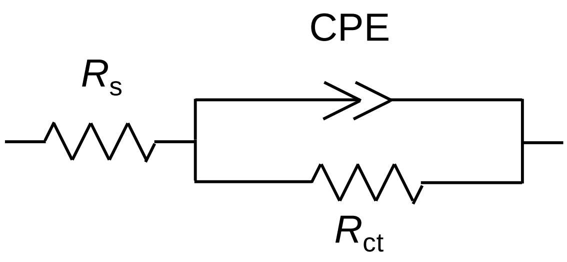

Figure S2. Equivalent circuit for fitting the recorded Nyquist plots. R_s: solution resistance; R_{ct}: charge-transfer resistance; CPE: constant phase element.

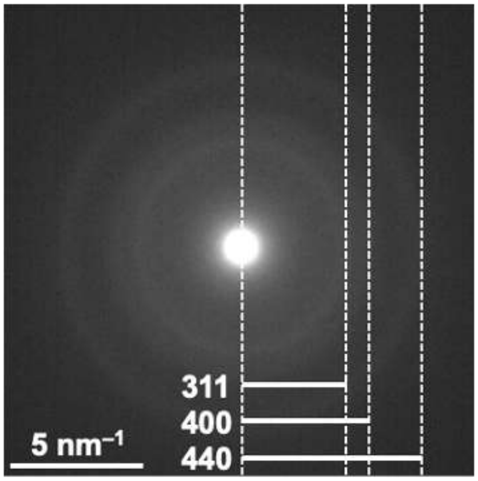

Figure S3. The SAED pattern of $NiMn_2O_4$ nanospinel.

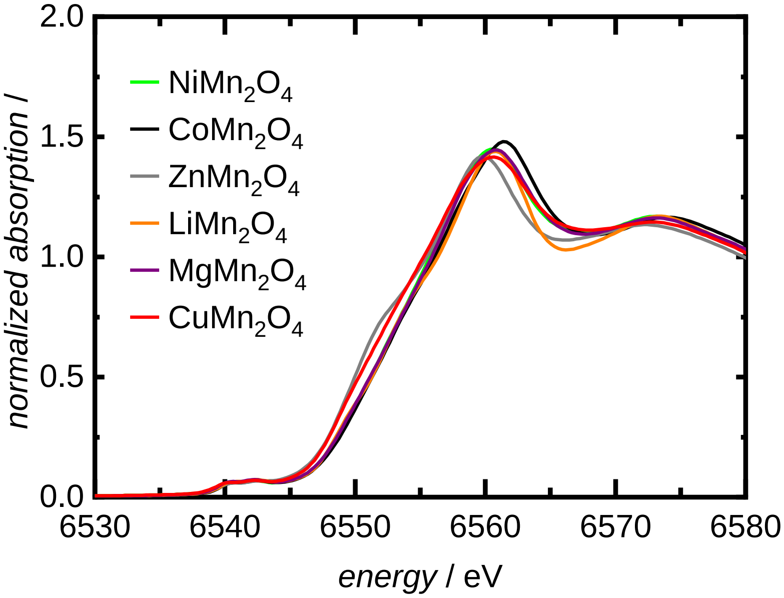

Figure S4. The Mn-K edge XANES spectra of the synthesized Mn-based nanospinels, which were recodred using their powder samples.

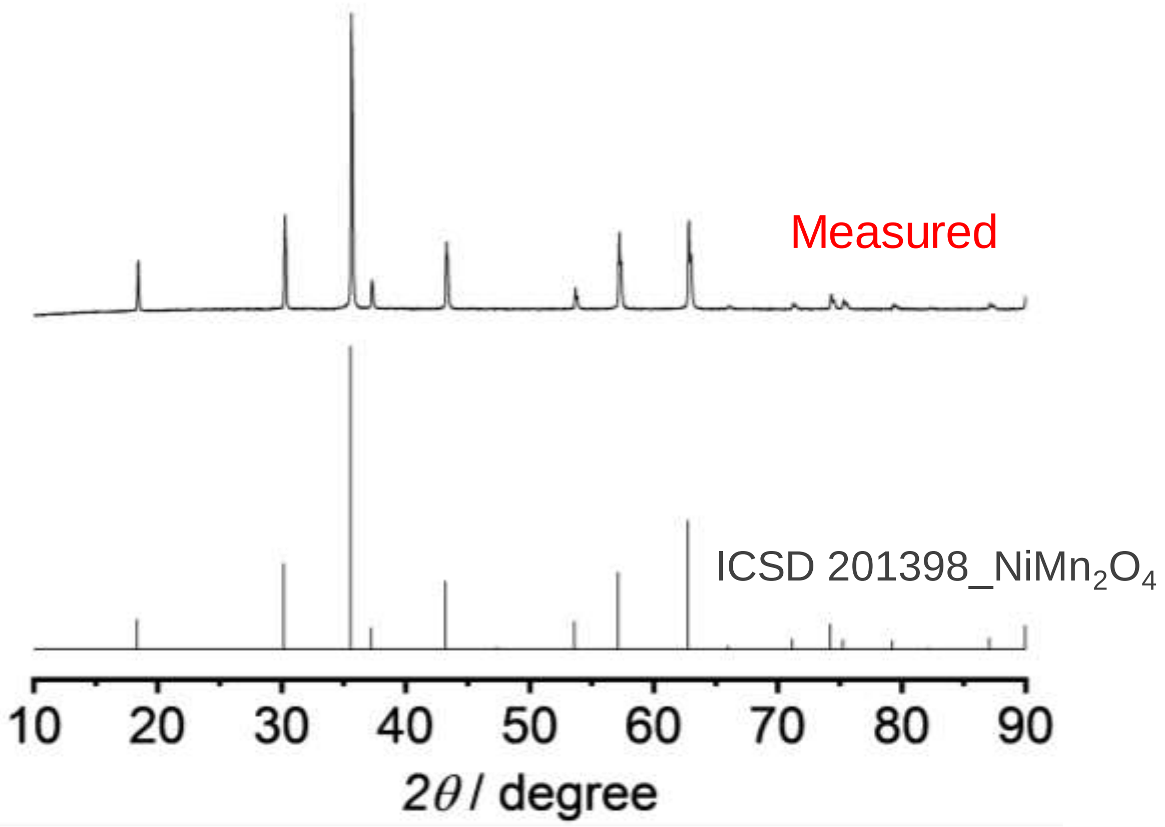

Figure S5. XRD patterns of (upper) measured $NiMn_2O_4$ normal spinel and (lower) theoretical $NiMn_2O_4$ spinel (ICSD: 201398).

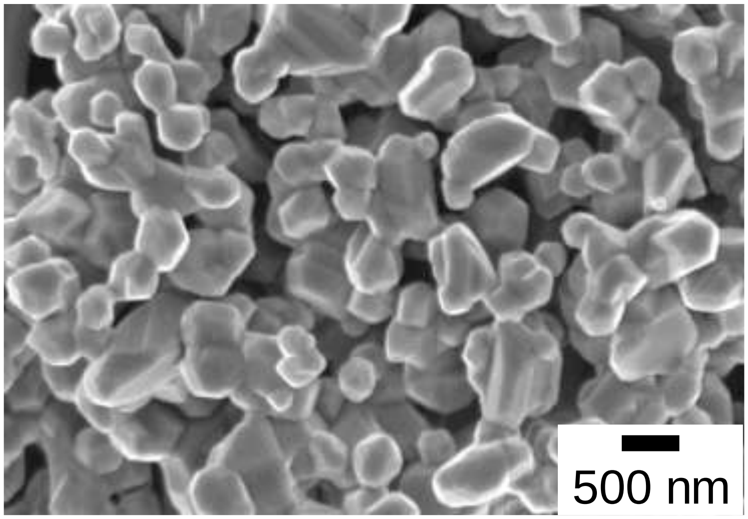

Figure S6. SEM image of synthesized $NiMn_2O_4$ normal spinel.

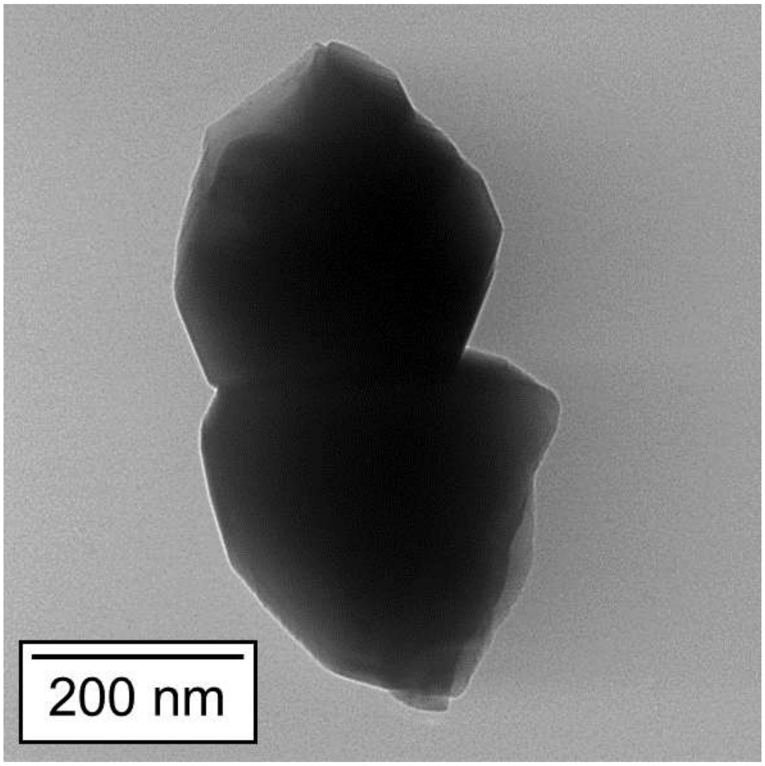

Figure S7. TEM image of synthesized $NiMn_2O_4$ normal spinel.

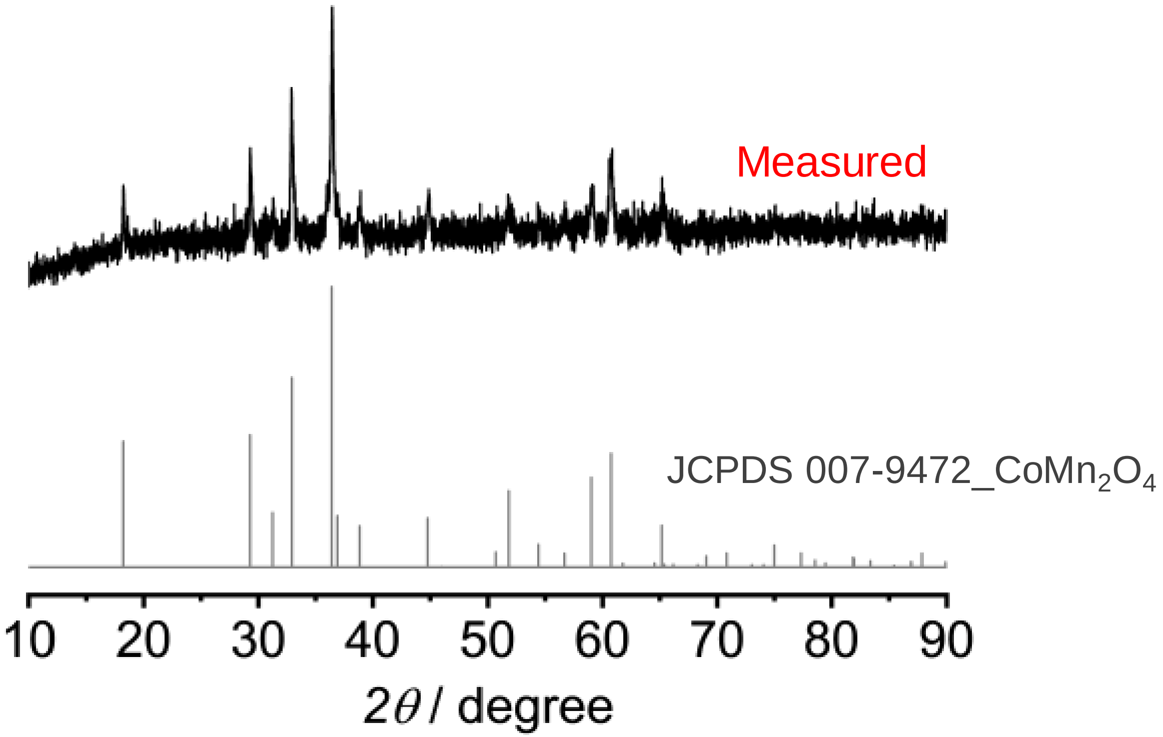

Figure S8. XRD patterns of (upper) measured $CoMn_2O_4$ normal spinel and (lower) theoretical $CoMn_2O_4$ spinel (JCPDS: 007-9472).

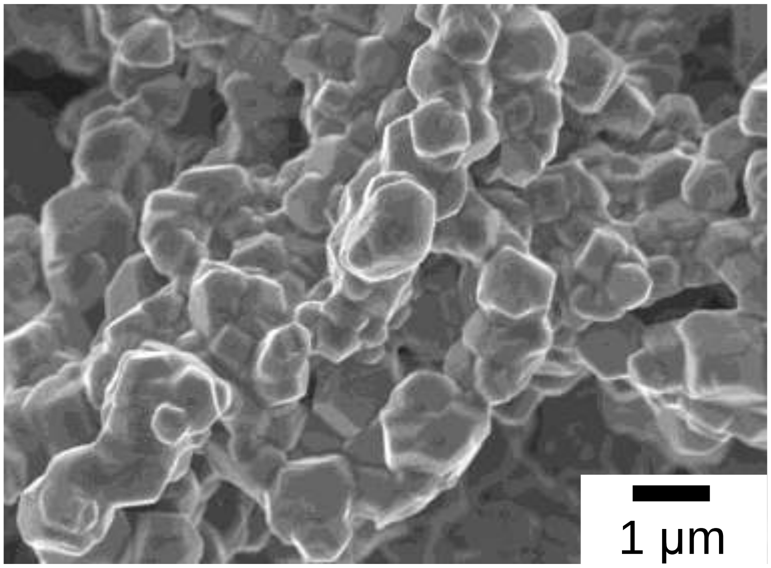

Figure S9. SEM image of synthesized $CoMn_2O_4$ normal spinel.

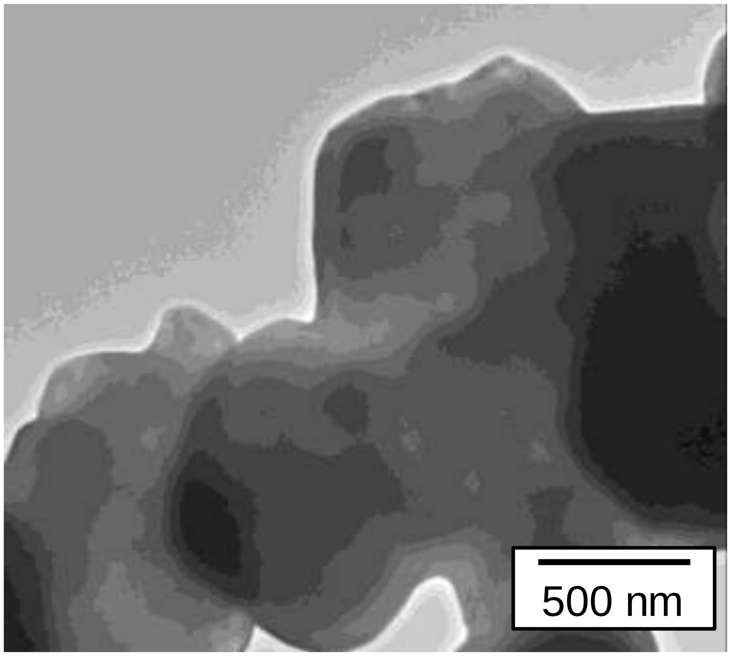

Figure S10. TEM image of synthesized $CoMn_2O_4$ normal spinel.

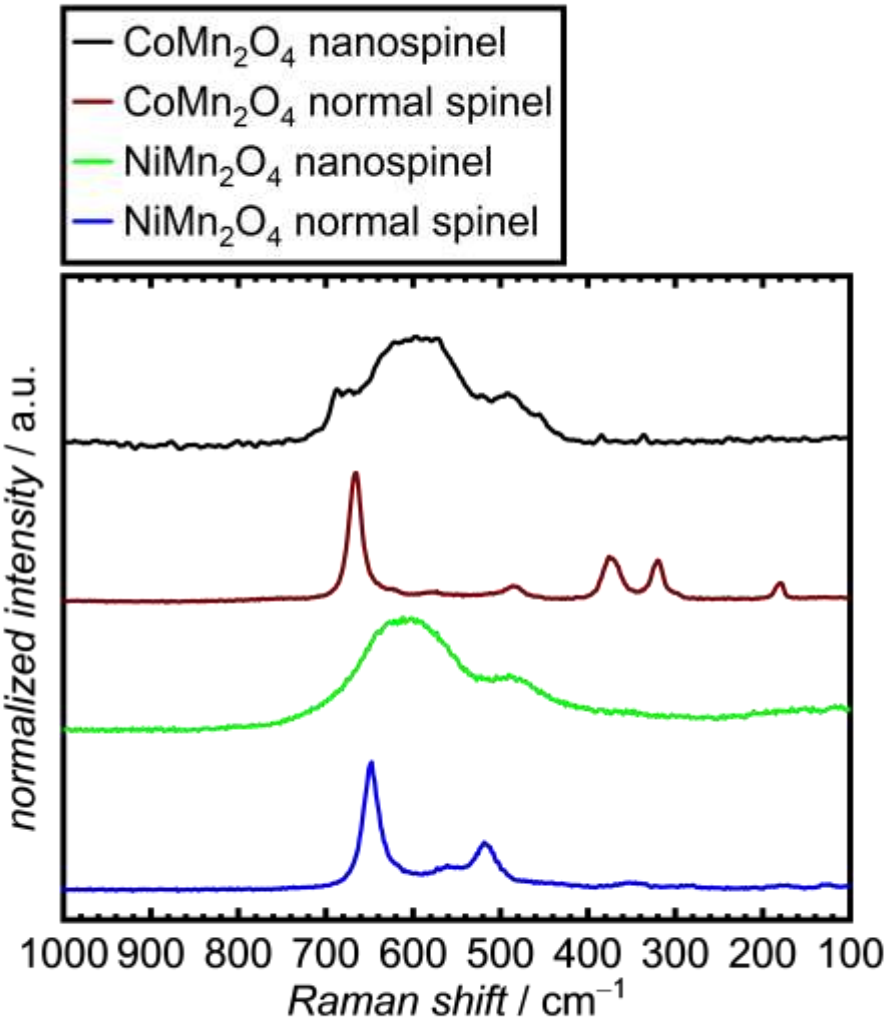

Figure S11. Comparison of Raman spectra for $NiMn_2O_4$ and $CoMn_2O_4$ nanospinels with normal spinels.

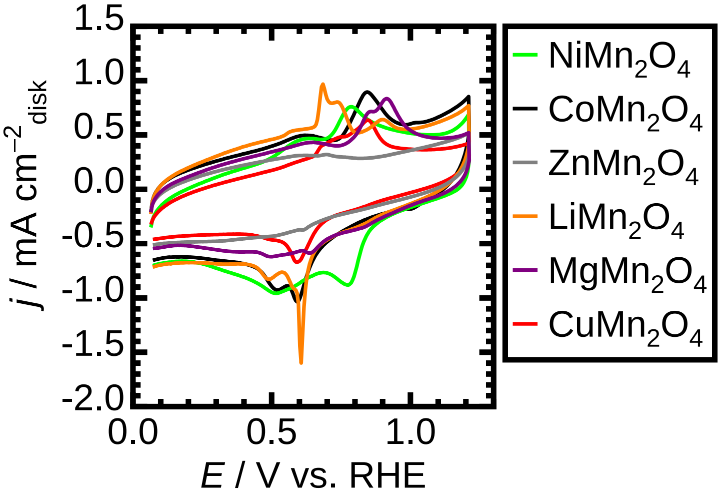

Figure S12. CV curves for nanospinels in N_2-saturated 1 M KOH.

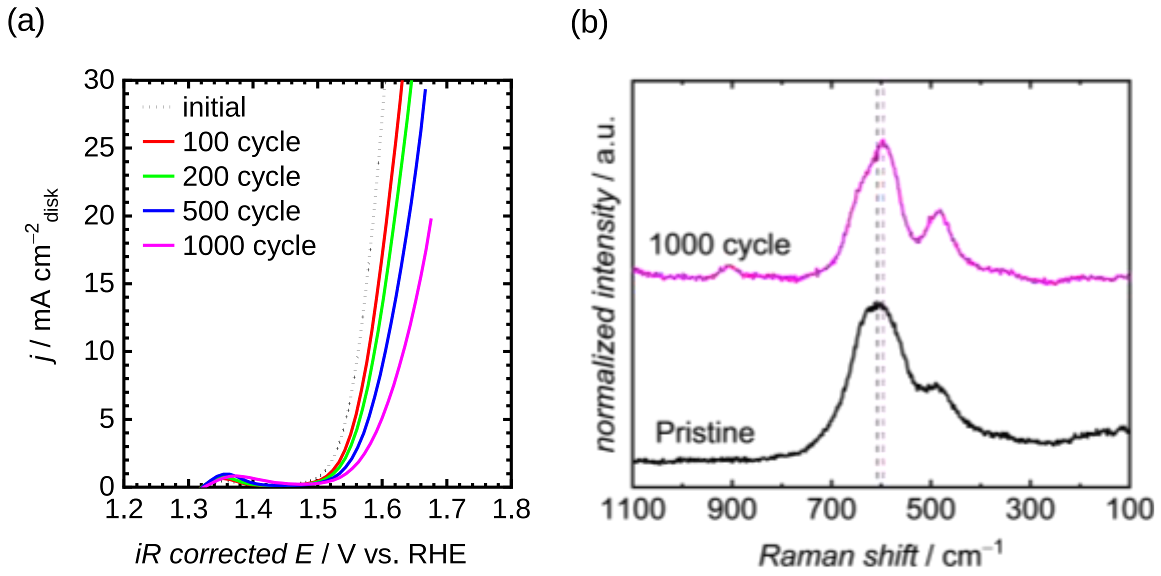

Figure S13. (a) Initial, 100th, 200th, 500th, and 1000th polarization curves of $NiMn_2O_4$ nanospinel in N_2-saturated 1 M KOH with a rotation speed of 1600 rpm. (b) Raman spectra of $NiMn_2O_4$ nanospinel before and after 1000 cycles.

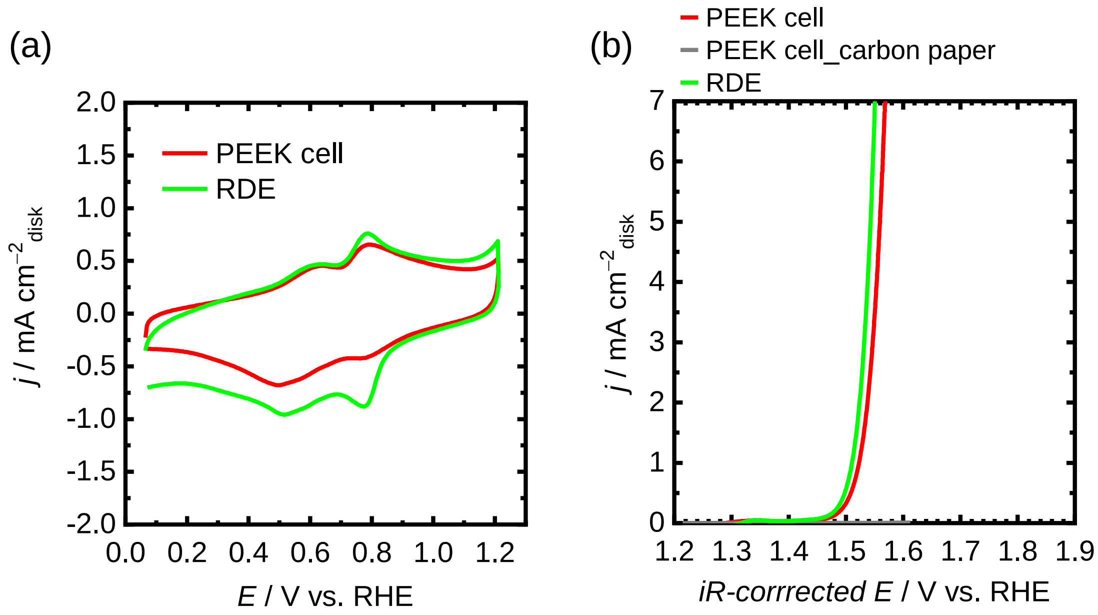

Figure S14. Comparison of electrochemical measurements for $NiMn_2O_4$ nanospinel using the PEEK cell with RDE. (a) CV pretreatment. (b) OER polarization curves. The measurements were carried out in O_2-saturated 1 M KOH. The RDE was rotated at 1600 rpm for OER polarization curve.

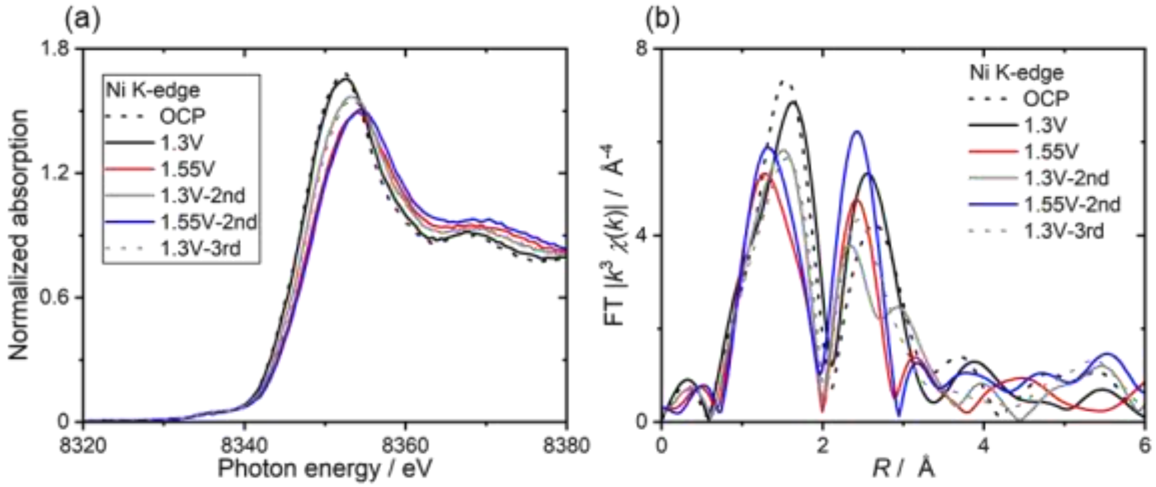

Figure S15. Ni-K edge XANES and EXAFS spectra for repeated-cycle measurements. (a) XANES and (b) EXAFS for $NiMn_2O_4$ nanospinel, respectively.

OCP: Open circuit potential.

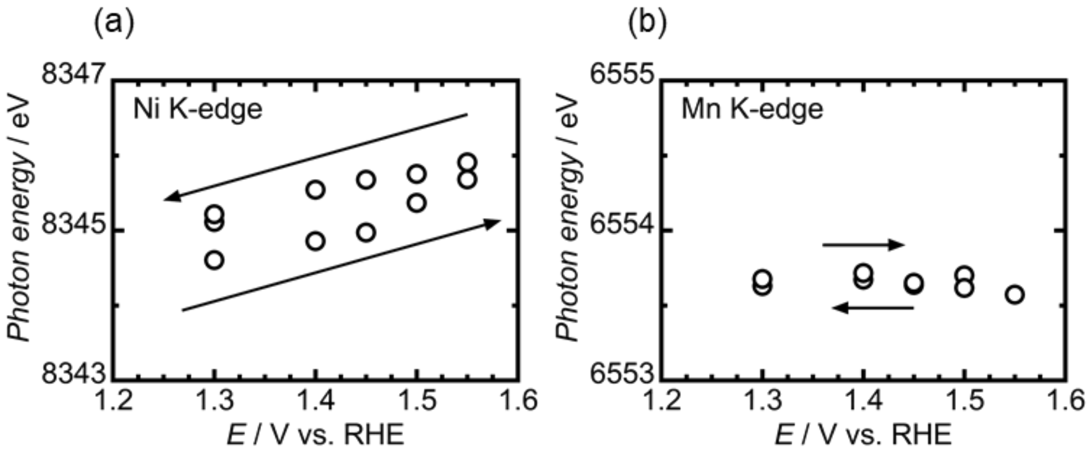

Figure S16. Shift of XANES spectra for (a) Ni K-edge and (b) Mn K-edge during the in-situ electrochemical measurements. The energy values were taken from the mid-point of the white line of XANES spectra.

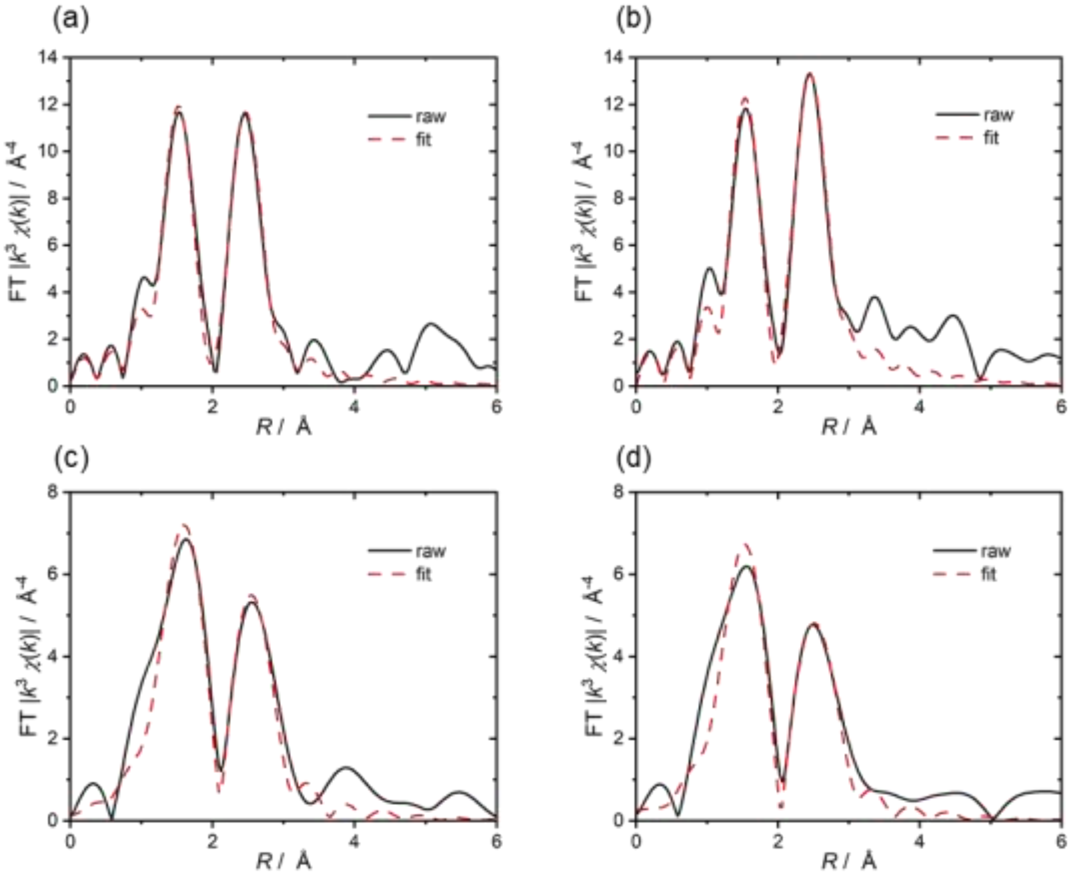

Figure S17 Representative FT-EXAFS spectra and curve-fittings under the potential applications for $NiMn_2O_4$ nanospinels (a-b) Mn K-edge, (c-d) Ni K-edge. The applied potential is 1.3 V vs. RHE for (a,c) and 1.4 V for (b,d).

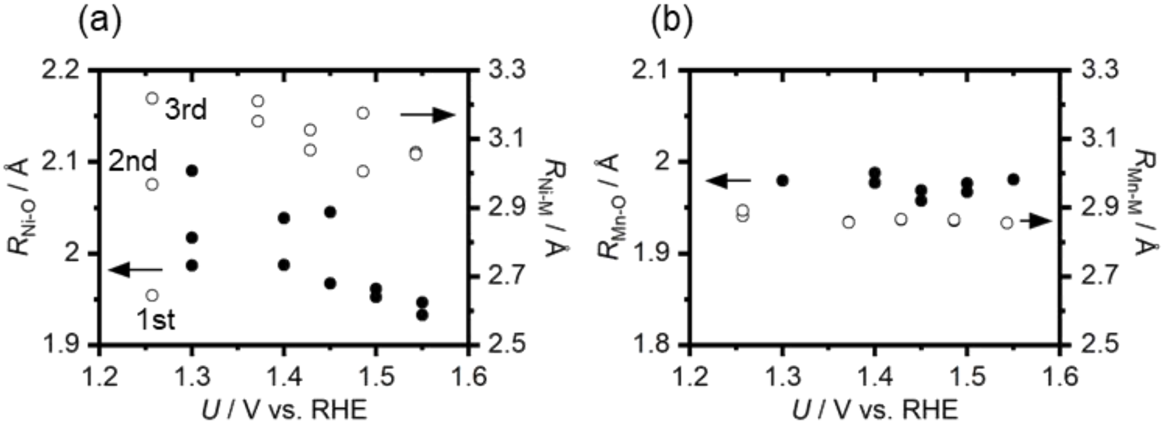

Figure S18. The EXAFS fitting results from electrochemical in-situ measurements showing the M–O bond distance (left) and M–M bond distance (right) as functions of the applied potential. (a) Ni K-edge and (b) Mn K-edge.

Table S1. Comparison of OER activities of $NiMn_2O_4$ with previously reported bimetal spinel oxides.

Catalyst	Overpotential [V] @10 mA/cm^2	Ref.
$NiMn_2O_4$ nanospinel	0.33	This study
$NiMn_2O_4$ (normal spinel)	0.38	This study
$MnCo_2O_4$	0.29	[1]
$NiCo_2O_4$	0.29	[2]
$ZnCo_2O_4$	0.39	[3]
$CoFe_2O_4$	0.34	[4]
$FeCo_2O_4$	0.48	[5]
$CoMn_2O_4$	0.38	[6]
$NiFe_2O_4$	0.33	[7]
$Li_{0.5}CoO_2$	0.35	[8]

Table S2. The results of fitting analyses for Mn K-edge EXAFS.

Potential	Mn-O			Mn-M[a]			R-factor
	R / Å	σ^2 / Å^2	CN[b]	R / Å	σ^2 / Å^2	CN[b]	
1.3 V	1.93 ± 0.01	0.003 ± 0.001	6.7 ± 1.0	2.88 ± 0.01	0.003 ± 0.001	7.0 ± 1.4	0.016
1.4 V	1.94 ± 0.02	0.002 ± 0.002	5.9 ± 1.5	2.86 ± 0.02	0.001 ± 0.001	6.4 ± 1.3	0.040
1.45 V	1.91 ± 0.02	0.005 ± 0.002	8.3 ± 1.7	2.86 ± 0.01	0.001 ± 0.002	5.7 ± 1.6	0.027
1.5 V	1.93 ± 0.01	0.004 ± 0.002	7.1 ± 1.2	2.86 ± 0.01	0.004 ± 0.002	8.3 ± 1.8	0.021
1.55 V	1.93 ± 0.01	0.006 ± 0.002	7.4 ± 1.3	2.86 ± 0.01	0.001 ± 0.001	6.5 ± 1.3	0.017
1.5 V	1.92 ± 0.01	0.005 ± 0.002	8.1 ± 1.3	2.86 ± 0.01	0.003 ± 0.001	7.3 ± 1.5	0.017
1.45 V	1.92 ± 0.01	0.003 ± 0.002	6.8 ± 1.2	2.87 ± 0.01	0.001 ± 0.002	5.8 ± 1.4	0.020
1.4 V	1.93 ± 0.01	0.002 ± 0.002	6.3 ± 1.4	2.86 ± 0.02	0.001 ± 0.002	5.2 ± 1.8	0.035
1.3 V	1.93 ± 0.01	0.002 ± 0.001	6.6 ± 1.1	2.89 ± 0.01	0.002 ± 0.002	4.6 ± 1.1	0.019

a: Calculated using the Mn-Mn path.

b: Coordination number (CN)

σ^2: Debye–Waller factor

Table S3. The results of fitting analyses for Ni K-edge EXAFS.

Potential	Ni-O			Ni-M[a]			R-factor
	R / Å	σ^2 / Å^2	CN[b]	R / Å	σ^2 / Å^2	CN[b]	
1.3 V	2.09 ± 0.04	0.013 ± 0.006	3.8 ± 1.6	3.22 ± 0.06	0.019 ± 0.01	6.16 ± 5.33	0.043
1.4 V	2.04 ± 0.03	0.013 ± 0.005	3.4 ± 1.4	3.21 ± 0.06	0.022 ± 0.01	7.37 ± 6.12	0.042
1.45 V	2.05 ± 0.05	0.015 ± 0.008	3.6 ± 1.9	3.13 ± 0.08	0.016 ± 0.01	3.95 ± 4.32	0.058
1.5 V	1.96 ± 0.06	0.021 ± 0.010	5.0 ± 3.5	3.17 ± 0.07	0.015 ± 0.01	2.84 ± 2.98	0.077
1.55 V	1.95 ± 0.06	0.019 ± 0.011	4.2 ± 3.2	3.06 ± 0.09	0.013 ± 0.01	2.22 ± 2.99	0.090
1.5 V	1.95 ± 0.06	0.018 ± 0.011	4.3 ± 3.3	3.01 ± 0.09	0.009 ± 0.01	1.54 ± 2.21	0.079
1.45 V	1.97 ± 0.04	0.012 ± 0.006	2.8 ± 1.4	3.07 ± 0.06	0.012 ± 0.01	2.58 ± 2.23	0.044
1.4 V	1.99 ± 0.03	0.012 ± 0.005	2.6 ± 1.1	3.15 ± 0.05	0.018 ± 0.01	4.92 ± 3.14	0.036
1.3 V 2nd	1.99 ± 0.04	0.015 ± 0.007	4.1 ± 2.0	2.64 ± 0.06	0.039 ± 0.01	5.87 ± 5.07	0.059
1.55 V 2nd	1.93 ± 0.05	0.014 ± 0.008	3.5 ± 2.2	3.05 ± 0.06	0.010 ± 0.01	2.30 ± 2.14	0.060
1.3 V 3rd	2.02 ± 0.03	0.012 ± 0.004	2.9 ± 0.9	2.97 ± 0.05	0.030 ± 0.01	6.95 ± 4.38	0.031

a: Calculated using the Ni–Ni path.

b: Coordination number (CN)

σ^2: Debye–Waller factor

References

(1) Sun, C. C.; Yang, J.; Dai, Z. Y.; Wang, X. W.; Zhang, Y. F.; Li, L. Q.; Chen, P.; Huang, W.; Dong, X. C. Nanowires Assembled from $MnCo_2O_4$@C Nanoparticles for Water Splitting and All-Solid-State Supercapacitor. *Nano Res.* **2016**, *9*, 1300-1309.

(2) Gao, X. H.; Zhang, H. X.; Li, Q. G.; Yu, X. G.; Hong, Z. L.; Zhang, X. W.; Liang, C. D.; Lin, Z. Hierarchical $NiCo_2O_4$ Hollow Microcuboids as Bifunctional Electrocatalysts for Overall Water-Splitting. *Angew. Chem. Int. Ed.* **2016**, *55*, 6290-6294.

(3) Kim, T. W.; Woo, M. A.; Regis, M.; Choi, K. S. Electrochemical Synthesis of Spinel Type $ZnCo_2O_4$ Electrodes for Use as Oxygen Evolution Reaction Catalysts. *J. Phys. Chem. Lett.* **2014**, *5*, 2370-2374.

(4) Geng, J.; Kuai, L.; Kan, E. J.; Wang, Q.; Geng, B. Y. Precious-Metal-Free Co-Fe-O/rGO Synergetic Electrocatalysts for Oxygen Evolution Reaction by a Facile Hydrothermal Route. *ChemSusChem* **2015**, *8*, 659-664.

(5) Yan, W. N.; Yang, Z. R.; Bian, W. Y.; Yang, R. Z. $FeCo_2O_4$/Hollow Graphene Spheres Hybrid with Enhanced Electrocatalytic Activities for Oxygen Reduction and Oxygen Evolution Reaction. *Carbon* **2015**, *92*, 74-83.

(6) Sugawara, Y.; Kobayashi, H.; Honma, I.; Yamaguchi, T. Effect of Metal Coordination Fashion on Oxygen Electrocatalysis of Cobalt–Manganese Oxides. *ACS Omega* **2020**, *5*, 29388–29397.

(7) Li, P. X.; Ma, R. G.; Zhou, Y.; Chen, Y. F.; Liu, Q.; Peng, G. H.; Liang, Z. H.; Wang, J. C. Spinel Nickel Ferrite Nanoparticles Strongly Cross-Linked with Multiwalled Carbon Nanotubes as a Bi-Efficient Electrocatalyst for Oxygen Reduction and Oxygen Evolution.

RSC Adv. **2015**, *5*, 73834-73841.

(8) Maiyalagan, T.; Jarvis, K. A.; Therese, S.; Ferreira, P. J.; Manthiram, A. Spinel-Type Lithium Cobalt Oxide as a Bifunctional Electrocatalyst for the Oxygen Evolution and Oxygen Reduction Reactions. *Nat. Commun.* **2014**, *5*, 3949.
